# Development and validation of questionnaire to assess exposure of children to enteric infections in the rural northwest Ethiopia

**DOI:** 10.1038/s41598-022-10811-x

**Published:** 2022-04-25

**Authors:** Zemichael Gizaw, Alemayehu Worku Yalew, Bikes Destaw Bitew, Jiyoung Lee, Michael Bisesi

**Affiliations:** 1 Department of Environmental and Occupational Health and Safety, Institute of Public Health, College of Medicine and Health Sciences, University of Gondar, Gondar, Ethiopia; 2 Addis Continental Institute of Public Health, Addis Ababa, Ethiopia; 3 Global One Health Initiative (GOHi), the Ohio State University, Columbus, OH USA; 4 School of Public Health, Addis Ababa University, Addis Ababa, Ethiopia; 5 Division of Environmental Health Sciences, College of Public Health, The Ohio State University, 1841 Neil Avenue, Columbus, OH 43210 USA; 6 Department of Food Science and Technology, The Ohio State University, Columbus, OH USA

**Keywords:** Microbiology, Diseases

## Abstract

In areas where children have multiple environmental exposures to enteric pathogens, identifying the sources of exposure by measuring external and internal exposures to enteric pathogens and complementing by questionnaire and observational checklist to capture behaviors resulting risk of exposure is critical. Accordingly, this study was conducted to design valid and reliable questionnaire to assess behaviors and environmental conditions resulting exposure to enteric pathogens in the rural northwest Ethiopia. We began with a thorough exploration of relevant literature to understand the theoretical framework on the research objectives to identify variables to highlight what the questionnaire is measuring. We then generated items in each domain that can effectively address the study objectives and we refined and organized the items in a suitable format. Then after, we conducted face and content validity by involving experts on the research subject. After pre-testing a pre-final version of the instrument generated in the content validity study, we conducted a pilot study in 150 randomly selected rural households to test the internal consistency reliability. We used content validity ratio (CVR), item-level content validity index (I-CVIs), scale-level content validity index (S-CVI/UA), and modified kappa statistics to measure content validity of items. Moreover, we used agreement and consistency indices (i.e., Cronbach’s alpha) to assess the internal consistency of items. The content validity test result showed that the value of CVR was 0.95, I-CVIs was 0.97, and modified kappa was 0.97 for the whole items, indicating all the items are appropriate. The scale-level content validity index (S-CVI/UA) was 0.95 for the whole items indicating the agreement among judges to each items is higher. The internal consistency reliability test result indicated that Cronbach’s alpha for the pre-final version of the pre-final tool was 0.85, indicating the strong reliability of the tool. The final version of the questionnaire was, therefore, prepared with 8 dimensions and 80 items. In this study, we designed valid and reliable questionnaire to assess behaviors and environmental conditions that result high risk of exposure to enteric infections in rural settings. The questionnaire can be used as a tool in the rural settings of developing countries with some amendments to account local contexts. However, this questionnaire alone does not measure exposure of children to enteric infections. It only complements external and internal exposure assessments.

## Introduction

The home environment can act as a reservoir for microbial colonization and can contribute to the spread of infectious diseases. Poor water, sanitation and hygiene (WASH) leads to fecal contamination of the home environment, which increases the risk of disease transmission^1,2^. Children in low-resource settings experience to a variety of enteropathogen risk factors from various sources and exposure pathways (e.g., water, soil, food, hands, flies, and containers)^1^. Similarly, inadequate access to basic sanitation, poor animal husbandry or keeping practice, and mouthing of soil contaminated materials are the commonest risk factors for transmission of enteric infections among children in the rural Ethiopia. A study done to measure child exposure to enteric infection in the rural northwest Ethiopia showed that contamination of water, food, and soil with fecal matter due to open defecation practice and poor animal keeping practice was common. Moreover, mouthing of soil or soil contaminated materials is commonly practiced among children in the rural Ethiopia^3^.

Various approaches for measuring human exposure exist along the environmental exposure pathway continuum, ranging from those that measure environmental contaminants to predict exposures before the contaminant reaches the human boundary (external exposure assessment) to those that estimate a dose after the contaminant has been taken up into the body (internal exposure assessment) (internal exposure assessment). The detection of indicators of fecal contamination or specific pathogens in a known size of environmental sample is a common approach for external exposure assessments^4^. Internal enteric pathogen exposure assessments using human biological specimens, on the other hand, can estimate actual enteric pathogen exposure after crossing the human body, typically through oral ingestion^4,5^.

Moreover, survey questionnaire can complement exposure assessments and the analysis of exposure data. Survey data on self-reported behaviors has been used as a rapid and cost-effective tool to collect information on a range of self-reported behaviors that result high risk of exposure^6^. However, questionnaire may result biased outputs unless it is valid and reliable. The quality of survey questionnaire is mostly related to the validity and reliability of the data obtained from it. An instrument would be considered a good measure when it passes the tests of validity and reliability^7^. The purpose of this work is, therefore, to design valid and reliable questionnaire to assess behaviors and environmental conditions that result exposure of children to enteric infections in the rural northwest Ethiopia.

Validity is the degree to which the questionnaire measures what is intended to be measured. In the literature, several types of validity have been described^8–10^. In this work, we included only face validity and content validity. Face validity is established when experts on the research subject reviewing the questionnaire concludes that it measures the research question/s^11,12^. When an expert examines the items in a questionnaire and agrees that the test is a valid measure of the concept being measured, this is known as face validity^13^. Content validity is the degree to which the questionnaire fully assesses the research question/s and it is achieved by a rational analysis of the instrument by experts on the research subject^13–15^.

Reliability is the degree to which a questionnaire produces consistent results over time. It refers to the consistency of scores over time or between raters. A pilot test is usually used to determine the questionnaire's reliability. Test–retest reliability, alternate form reliability, and internal consistency reliability are the three major types of reliability that can be assessed^16^. In this work, we used internal consistency reliability to assess the reliability of our questionnaire.

## Methods

**Figure 1:**
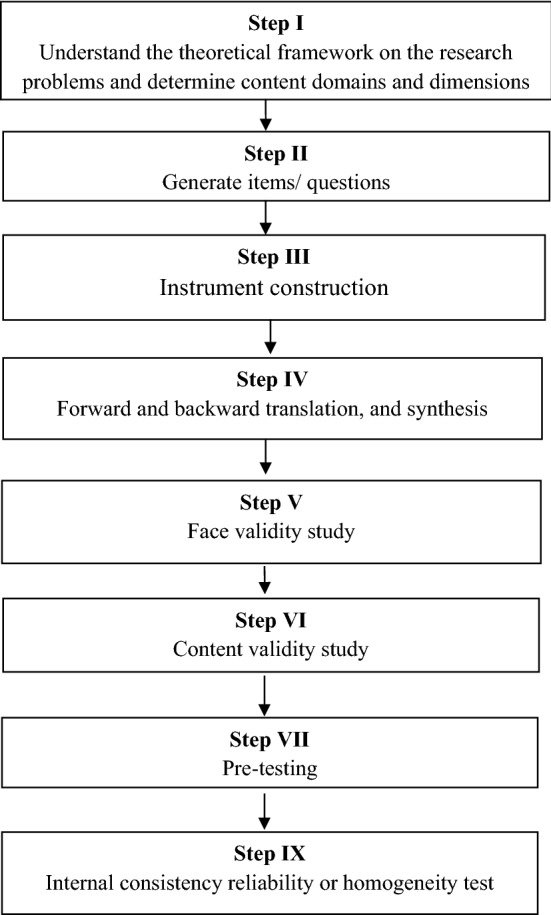
Steps of tool development and validation study.

We used a method described by Zamanzadeh V, et al.^17^ to design the questionnaire and to test its validity and internal consistency. Our work first describes the steps involved in the design of questionnaire and the procedures of testing validity and reliability of the instrument (Fig. 1).

### Step I: Understanding the theoretical framework on the research problems and determine content domains

**Figure 2:**
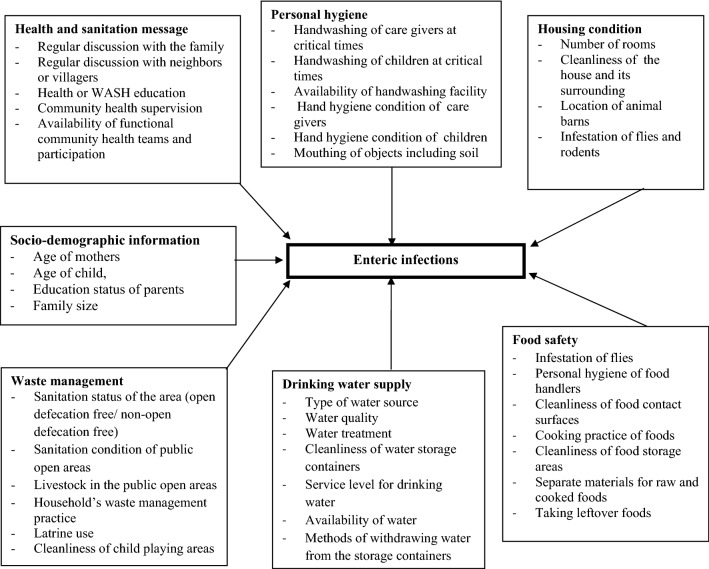
Content domains and variables that the questionnaire will measure.

In this step, we began with a thorough exploration of relevant literature to understand the theoretical framework on the research problems and objectives to determine content domains and to identify major variables to highlight what the questionnaire is measuring. Figure 2 summarizes the content domains and variables that the questionnaire will measure.

### Step II: Item generation

We generated items/questions in each domain that can effectively address the study objectives or research questions after developing a good understanding of the theoretical framework on the research problems and objectives through review of literature. Each item in the questionnaire was generated based on the content domains and variables summarized in Fig. 2.

### Step III: Instrument construction

In this step, we refined and organized the items in a suitable format and sequence so that the finalized items are collected in a usable form. Research team members reviewed and approved the final preliminary version of the instrument.

### Step IV: Forward and backward translation and synthesis

The questionnaire, which was written in English, was translated into Amharic, the local language. Two native Amharic speakers fluent in English independently completed the translation, which was then back-translated into English by two independent English language experts fluent in Amharic who were blinded to the English version. The back-translated versions were checked for discrepancies against the original version. The preliminary version was ready for face validity after discrepancies were corrected.

### Step V: Face validity

After translation, we conducted a face validity study by involving 12 experts on the field who are working at the University of Gondar, Ethiopia (3 environmental health experts, 4 microbiologists, 3 pediatric nurses, and 2 nutritionists). All the experts had 10 or above years of experience. All the environmental health experts and microbiologists were PhD holders and the rest experts were second degree holder. We dispatched the Amharic version questionnaire attached with a conceptual framework and study objectives to these experts to review it critically with clear instruction and we arranged a panel discussion after a week to discuss on each item in the questionnaire line-by-line and to collect and analyze their quantitative and qualitative viewpoints on the relevancy or representativeness, clarity and comprehensiveness of the items. In the discussion, experts evaluated the questions whether they appropriately measure the research objectives or not. Experts also added some relevant questions that can answer the study objectives and removed some questions that have little contributions to the study objectives. Experts also judged the way the questions were organized and acceptability of the questionnaire by study participants^18^.

### Step VI: Content validity

After the face validity, we dispatched the questionnaire developed in the face validity attached with the conceptual framework and study objectives to 35 experts on the research subject. The experts were selected based on the following criteria: (i) area of expertise (environmental health, microbiology, parasitology, epidemiology, nutrition, and pediatric nurse); (ii) year of experience (10 or above years of experience); (iii) level of education (second degree or above); and (iv) research experience (assistant professor or above). Experts had been told to critically review the questionnaire line-by-line by referring the study objectives and conceptual framework and to rate the degree to which the questionnaire fully assesses or measures the study objectives. We told experts to rate the relevance of items in the questionnaire as ‘not relevant’ (which is assign a score of 1), ‘somehow relevant’ (which is assign a score of 2), ‘quite relevant’ (which is assign a score of 3), and ‘highly relevant’ (which is assign a score of 4)^17^.

To select the most important and correct item in the instrument, we calculated a content validity ratio (CVR). The experts' scores were used to calculate the CVR for each questionnaire item using the Lawshe method^19^.

$\mathrm{CVR}= \frac{{(\mathrm{N}}_{\mathrm{e}} -\mathrm{ N}/2)}{\mathrm{N}/2}$, where Ne is the number of experts indicating the item is essential and N is the total number of experts. If CVR is bigger than 0.49, the item in the instrument with an acceptable level of significance will be accepted^19^.

Item-level content validity index (I-CVIs) was used to determine the proportion of agreement on the relevancy or appropriateness of each item. I-CVI is computed as the number of experts giving a rate of 3 or 4 to the relevancy of each item, divided by the total number of experts^17^. After calculating I-CVI, judgment on each item is made as follows: If the I-CVI is higher than 79%, the item is appropriate. If it is between 70 and 79%, it needs revision. If it is less than 70%, it is eliminated^20^. To determine the proportion of total items judged content valid, we used Scale-level content validity index (S-CVIs) which we calculated using universal agreement approach (S-CVI/UA) among experts. We first dichotomized the scale in to ‘relevant’ by combining values 3 and 4 together and ‘not relevant’ by combining values 2 and 1 together and then, the number of items considered ‘relevant’ is divided by the total number of items^21,22^. For the S-CVI/UA, 80% agreement or higher among judges was considered^22^.

Moreover, CVI does not consider the possibility of inflated values because of the chance agreement; we used both CVI and multi-rater kappa statistic to adjust for chance agreement^23,24^. To calculate modified kappa statistics, the probability of chance agreement, was first calculated for each item by following formula^17^:

$${\mathrm{P}}_{\mathrm{c}}=[\mathrm{N}!/\mathrm{A}!(\mathrm{N}-\mathrm{ A})!]*0.{5}^{\mathrm{N}}$$

where N = number of experts in a panel and A = number of panelists who agree that the item is relevant. After calculating I-CVI for all instrument items, finally, kappa was computed by entering the numerical values of probability of chance agreement (PC) and content validity index of each item (I-CVI) in following formula^24^:

$$\mathrm{K}= \frac{\mathrm{I}\_\mathrm{CVI }- {\mathrm{P}}_{\mathrm{c}}}{1-{\mathrm{P}}_{\mathrm{c}}}$$

Kappa values above 0.74 are considered as excellent, between 0.60 and 0.74 as good, and between 0.40 and 0.59 are considered as fair^25^.

### Step VII: Pre-testing

The instrument generated in the content validity study was pre-tested among 10 selected rural households having similar characteristics to the target population in which the instrument will be used to evaluate the instructions, response format and the items of the instrument for clarity and a pre-final version of the instrument was generated.

### Step VIII: Internal consistency reliability test

A pilot study in 150 randomly selected rural households was undertaken using the pre-final version of the instrument to test the internal consistency reliability. The minimum sample size (i.e., 150) for the internal consistency reliability study was determined based on the recommendations in the literature^26–28^. The pilot study was conducted in the rural setting of the east Dembiya district of Ethiopia in December 2020. The east Dembiya district is one of the districts in central Gondar zone, the Amhara national regional state, Ethiopia. As of July 2020, the district had a total of 192,020 rural and 18,741 urban residents^29^, of these, 39,927 (12.22%) were children under age five-years^30^. In the district, coverage of clean water and latrine in 2017 were 26.6% and 55%, respectively and the households are not linked to municipal water and sewage system in the area. Moreover, during June 2017, intestinal parasitic infections and diarrheal disease were the top four and five prevalent, which accounted 5161 (9.97%) and 4981 (9.62%), respectively. In the area domestic animals and their feces are not properly contained or separated from the living environments^31^.

All households in the rural kebeles (the lowest administrative unit in Ethiopia) in the district were considered for sampling. First, we chose three rural kebeles at random out of 28 kebeles using a simple random sampling technique. We allocated equal number of households with children under the age of five-years to each kebele. Finally, 150 households were included in the study using a systematic random sampling technique.

Field data collectors interviewed the female head of the household to collect data using the pre-final version of the instrument. The collected data were entered to Epi Info version 7 and exported to Stata version 14 for analysis. We assessed reliability using agreement and consistency indices. Cronbach’s alpha was computed to assess the internal consistency of items^32,33^ and values of ≥ 0.70 were considered adequate^16^.

### Ethics approval and consent to participate

Ethical clearance was obtained from the Institutional Review Board of the University of Gondar (reference number: V/P/RCS/05/1933/2020). There were no risks due to participation and the collected data were used only for this research purpose with complete confidentiality. Written informed consent was obtained from study participants. All the methods were carried out in accordance with relevant guidelines and regulations.

### Consent for publication

This manuscript does not contain any individual person’s data.

## Results

### Identification of content domains and item generation

In the first step of instrument design, ten content domains including socio-demographic, health and sanitation messages, healthcare seeking behavior for childhood illnesses, personal hygiene, excreta management, water quality and safety measures, food hygiene and safety measures, housing conditions, infestation of vectors, and enteric infections were identified.

In the item generation step, 123 items were generated from these domains [10 from socio-demographic domain, 10 from health and sanitation messages domain, 11 from healthcare seeking behavior for childhood illnesses domain, 11 from personal hygiene domain, 17 from excreta management domain, 20 from water quality and safety measures domain, 18 from food hygiene and safety measures domain, 16 from housing conditions domain, 8 from enteric infections domain, and 2 from infestation of vectors domain]. We refined and organized all these items in a suitable format.

### Face validity

In the face validity study, experts re-categorized the content domains in to eight and added some relevant questions in each domain and removed some questions from each domain. Accordingly, 80 items [8 from socio-demographic domain, 8 from health and sanitation message domain, 12 from personal hygiene domain, 12 from waste management domain, 15 from drinking water supply domain, 11 from food safety domain, 8 from housing condition domain, and 6 from enteric infection domain] were generated.

### Content validity

We calculated CVR, I-CVI, S-CVI/UA, and modified kappa based on the formulas described in the method section. The CVR, I-CVI, and modified kappa values for the total items were 0.95, 0.97, and 0.97, respectively. Moreover the CVR, I-CVI, and modified kappa values for each item were greater than the cut values (0.49, 0.79, and 0.74, respectively), indicating that all the items generated in the face validity test are appropriate to measure the research objectives (Table 1).

**Table 1:** Instrument domains, total number of items, Item-level Content Validity Index, Modified Kappa, and interpretations in the content validity study [number of domains = 8, total number of items = 80, number of content experts = 35, cut point for CVR ≥ 0.49, cut point for I-CVI ≥ 0.79, and cut point for Modified Kappa ≥ 0.74].

Dimensions and items	Experts rated items as relevant	CVR	Interpretation	I-CVI	Interpretation	Modified Kappa	Interpretation
**Socio-demographic conditions**							
101	35	1	Remained	1	Appropriate	1	Excellent
102	35	1	Remained	1	Appropriate	1	Excellent
103	35	1	Remained	1	Appropriate	1	Excellent
104	35	1	Remained	1	Appropriate	1	Excellent
105	35	1	Remained	1	Appropriate	1	Excellent
106	35	1	Remained	1	Appropriate	1	Excellent
107	35	1	Remained	1	Appropriate	1	Excellent
108	35	1	Remained	1	Appropriate	1	Excellent
**Health and sanitation messages**							
201	34	0.94	Remained	0.97	Appropriate	0.97	Excellent
202	35	1	Remained	1	Appropriate	1	Excellent
203	35	1	Remained	1	Appropriate	1	Excellent
204	35	1	Remained	1	Appropriate	1	Excellent
205	33	0.89	Remained	0.94	Appropriate	0.94	Excellent
206	35	1	Remained	1	Appropriate	1	Excellent
207	35	1	Remained	1	Appropriate	1	Excellent
208	35	1	Remained	1	Appropriate	1	Excellent
**Personal hygiene**							
301	34	0.94	Remained	0.97	Appropriate	0.97	Excellent
302	34	0.94	Remained	0.97	Appropriate	0.97	Excellent
303	34	0.94	Remained	0.97	Appropriate	0.97	Excellent
304	34	0.94	Remained	0.97	Appropriate	0.97	Excellent
305	32	0.83	Remained	0.91	Appropriate	0.91	Excellent
306	34	0.94	Remained	0.97	Appropriate	0.97	Excellent
307	34	0.94	Remained	0.97	Appropriate	0.97	Excellent
308	35	1	Remained	1	Appropriate	1	Excellent
309	35	1	Remained	1	Appropriate	1	Excellent
310	34	0.94	Remained	0.97	Appropriate	0.97	Excellent
311	34	0.94	Remained	0.97	Appropriate	0.97	Excellent
312	35	1	Remained	1	Appropriate	1	Excellent
**Waste management**							
401	34	0.94	Remained	0.97	Appropriate	0.97	Excellent
402	35	1	Remained	1	Appropriate	1	Excellent
403	34	0.94	Remained	0.97	Appropriate	0.97	Excellent
404	33	0.89	Remained	0.94	Appropriate	0.94	Excellent
405	34	0.94	Remained	0.97	Appropriate	0.97	Excellent
406	35	1	Remained	1	Appropriate	1	Excellent
407	34	0.94	Remained	0.97	Appropriate	0.97	Excellent
408	33	0.89	Remained	0.94	Appropriate	0.94	Excellent
409	32	0.83	Remained	0.91	Appropriate	0.91	Excellent
410	31	0.77	Remained	0.89	Appropriate	0.89	Excellent
411	32	0.83	Remained	0.91	Appropriate	0.91	Excellent
412	30	0.71	Remained	0.86	Appropriate	0.83	Excellent
**Drinking water supply**							
501	32	0.83	Remained	0.91	Appropriate	0.91	Excellent
502	32	0.83	Remained	0.91	Appropriate	0.91	Excellent
503	35	1	Remained	1	Appropriate	1	Excellent
504	35	1	Remained	1	Appropriate	1	Excellent
505	31	0.77	Remained	0.89	Appropriate	0.89	Excellent
506	35	1	Remained	1	Appropriate	1	Excellent
507	32	0.83	Remained	0.91	Appropriate	0.91	Excellent
508	34	0.94	Remained	0.97	Appropriate	0.97	Excellent
509	30	0.71	Remained	0.86	Appropriate	0.83	Excellent
510	35	1	Remained	1	Appropriate	1	Excellent
511	35	1	Remained	1	Appropriate	1	Excellent
512	35	1	Remained	1	Appropriate	1	Excellent
513	34	0.94	Remained	0.97	Appropriate	0.97	Excellent
514	34	0.94	Remained	0.97	Appropriate	0.97	Excellent
515	35	1	Remained	1	Appropriate	1	Excellent
**Food safety**							
601	35	1	Remained	1	Appropriate	1	Excellent
602	35	1	Remained	1	Appropriate	1	Excellent
603	35	1	Remained	1	Appropriate	1	Excellent
604	35	1	Remained	1	Appropriate	1	Excellent
605	35	1	Remained	1	Appropriate	1	Excellent
606	33	0.89	Remained	0.94	Appropriate	0.94	Excellent
607	34	0.94	Remained	0.97	Appropriate	0.97	Excellent
608	35	1	Remained	1	Appropriate	1	Excellent
609	34	0.94	Remained	0.97	Appropriate	0.97	Excellent
610	34	0.94	Remained	0.97	Appropriate	0.97	Excellent
611	35	1	Remained	1	Appropriate	1	Excellent
**Housing condition**							
701	35	1	Remained	1	Appropriate	1	Excellent
702	35	1	Remained	1	Appropriate	1	Excellent
703	33	0.89	Remained	0.94	Appropriate	0.94	Excellent
704	34	0.94	Remained	0.97	Appropriate	0.97	Excellent
705	35	1	Remained	1	Appropriate	1	Excellent
706	34	0.94	Remained	0.97	Appropriate	0.97	Excellent
707	35	1	Remained	1	Appropriate	1	Excellent
708	35	1	Remained	1	Appropriate	1	Excellent
**Enteric infections**							
801	35	0.94	Remained	0.97	Appropriate	0.97	Excellent
802	35	0.95	Remained	1	Appropriate	0.97	Excellent
803	35	0.94	Remained	0.94	Appropriate	0.94	Excellent
804	35	0.95	Remained	1	Appropriate	1	Excellent
805	35	0.94	Remained	0.97	Appropriate	1	Excellent
806	35	0.95	Remained	0.94	Appropriate	0.94	Excellent

The S-CVI/UA value for the total items was 0.95 and the values to each item were greater than the cut value, i.e., 0.80 (Table 2), which showed that the proportion of total items judged content valid is within the acceptable range or the agreement among judges is higher.

**Table 2:** Number of items considered relevant by content experts, Scale-level Content Validity Index, and interpretation [total number of items = 80, number of content experts = 35, and cut point ≥ 0.80].

Content experts	Number of items considered relevant	S-CVI/UA	Interpretation
Content expert 1	79	0.9875	Valid
Content expert 2	79	0.9875	Valid
Content expert 3	76	0.95	Valid
Content expert 4	79	0.9875	Valid
Content expert 5	72	0.9	Valid
Content expert 6	72	0.9	Valid
Content expert 7	76	0.95	Valid
Content expert 8	76	0.95	Valid
Content expert 9	79	0.9875	Valid
Content expert 10	78	0.975	Valid
Content expert 11	78	0.975	Valid
Content expert 12	80	1	Valid
Content expert 13	77	0.9625	Valid
Content expert 14	80	1	Valid
Content expert 15	79	0.9875	Valid
Content expert 16	74	0.925	Valid
Content expert 17	76	0.95	Valid
Content expert 18	75	0.9375	Valid
Content expert 19	80	1	Valid
Content expert 20	74	0.925	Valid
Content expert 21	76	0.95	Valid
Content expert 22	76	0.95	Valid
Content expert 23	76	0.95	Valid
Content expert 24	76	0.95	Valid
Content expert 25	74	0.925	Valid
Content expert 26	75	0.9375	Valid
Content expert 27	74	0.925	Valid
Content expert 28	75	0.9375	Valid
Content expert 29	74	0.925	Valid
Content expert 30	74	0.925	Valid
Content expert 31	76	0.95	Valid
Content expert 32	76	0.95	Valid
Content expert 33	72	0.9	Valid
Content expert 34	74	0.925	Valid
Content expert 35	74	0.925	Valid

In all cases, no item was eliminated in the content validity process. So, our instrument was prepared with 8 dimensions and 80 items for internal consistency reliability.

### Internal consistency

A pilot survey was carried out among 150 rural households to measure the internal consistency reliability of the questionnaire. Table 3 shows information about the socio-demographic and WASH profile of the 150 households.

**Table 3:** Socio-demographic and WASH profile of the rural households participated in the pilot survey (n = 150) in the east Dembiya district, December 2020.

Sociodemographic and WASH variables	Frequency	Percent
**Family size**		
$\le$ 5	94	62.7
$>$ 5	56	37.3
**Education status of the household head (female head)**		
Can't read and write	68	45.3
Can read and write	14	9.3
Primary education	23	15.3
Secondary education	28	18.7
Certificate/diploma	17	11.3
**Defecation practice of household members**		
Open field	90	60.0
Traditional pit latrine	60	40.0
**How the household manage domestic waste water**		
Use soak pit	18	12.0
Disposed everywhere in the yard	319	88.0
**How the household manage rubbish**		
Open dumping	105	70.0
Burning	32	21.3
Burial	13	8.7
**Animal excreta in the living environment**		
Yes	120	80.0
No	30	20.0
**Drinking water sources**		
Ground water	110	73.3
Surface water	40	26.7
**Drinking water sources**		
Protected	80	53.3
Unprotected	70	46.7
**How far the water sources located from the dwelling**		
Within 1 km radius	118	78.7
More than 1 km away	32	21.3
**Always washed food utensils with soap or ash**		
Yes	135	90.0
No	15	10.0

An internal consistency reliability analysis was carried out on a survey questionnaire on environmental exposures of children to enteric infections comprising 80 items. The Cronbach’s α was used to measure the internal consistency of the scale items. For the whole scale, Cronbach’s α was 0.85 and ranged between 0.79 and 0.85 (Table 4) for the eight dimensions, indicating the strong reliability of the tool. Therefore, the final version of the questionnaire was prepared with 8 dimensions and 80 items. The final English (Supplementary File 1) and Amharic (Supplementary File 2) versions are included as supplementary materials.

**Table 4:** Dimension descriptions and scale reliability.

Dimensions	Cronbach’s α
Dimension 1: Socio-demographic conditions	0.81
Dimension 2: Health and sanitation message	0.80
Dimension 3: Personal hygiene	0.85
Dimension 4: Waste management	0.85
Dimension 5: Drinking water supply	0.79
Dimension 6: Food safety	0.82
Dimension 7: Housing condition	0.85
Dimension 8: Enteric infections	0.80
Whole scale	0.85

## Discussion

This study was conducted to design valid and reliable questionnaire to complement exposure assessment of children to enteric infections in the rural northwest Ethiopia. As presented in this paper, a questionnaire assessing behaviors that result exposure of children to enteric infections was developed with satisfactory validity and reliability. The 8-domains and 80 items adopted in this study are appropriate or relevant to capture behaviors that result exposure of children to enteric infections. The domains included in the final version of the tool were socio-demographic domain, health and sanitation messages domain, personal hygiene domain, waste management domain, drinking water supply domain, food safety domain, housing condition domain, and enteric infection domain. These domains, as represented by the respective items per domain, appeared to be important. The content domains included in the final version of the questionnaire are partly or fully used in other studies to collect data on self-reported behaviors or observational data on practices to enable the targeting of environmental media and locations where the study population is predominantly exposed to enteric infections. The SaniPath tool is the standard tool researchers commonly used to complement external assessment^34–36^ and some studies combined external assessment with behavioral observations to estimate actual ingestion (e.g., measuring pathogens in soil and frequency of geophagia or measuring fecal indicators deposited by flies when alighting on food and the number of fly landings). However, these methods rely heavily on assumptions about conditions and behaviors that vary significantly within and between individuals^36^. Designing valid and reliable data collection tool that consider the local contexts in which the study will be conducted is very useful. This tool will be, therefore, used in the rural settings of developing countries to measure behaviors that result high exposure to enteric infections.

The CVR, I-CVI, and modified kappa for the total items and for each item were high, indicating that the items are appropriate to measure the research objectives. The S-CVI was also high for the total items and for each item, indicating the agreement among judges to each item is higher. CVR is an empirical analysis, which measures the essentiality of an item. CVR varies between 1 and -1, and a higher score indicates greater agreement among panel members^17^. I-CVI and S-CVI are the most widely reported approach for content validity. Values of I-CVI range from 0 to 1 where I-CVI > 0.79, the item is relevant, between 0.70 and 0.79, the item needs revisions, and if the value is below 0.70 the item is eliminated^17^. Eighty percent or higher values for S-CVI/UA is considered for acceptable agreement among judges^22^. The multi-rater kappa statistic adjusts chance agreement, whereas I-CVI and S-CVI do not consider the possibility of inflated values because of the chance agreement. Thus, checking the Kappa values to each item is important in addition to CVR, I-CVI, and S-CVI. Kappa values above 0.74 are considered as excellent, between 0.60 and 0.74 as good, and between 0.40 and 0.59 are considered as fair^25^.

The Cronbach’s α for the total scale was high (0.85) and all items appeared to be worthy of retention, resulting in a decrease in the alpha if deleted. The reliability coefficient (alpha) can range from 0 to 1, with 0 representing a questionnaire that is not reliable and 1 representing absolutely reliable questionnaire. Cronbach’s α coefficients $\ge$ 0.9 indicate excellent internal consistency, 0.8 > α ≥ 0.9 are good, 0.8 > α ≥ 0.7 are acceptable, 0.7 > α ≥ 0.6 are questionable, 0.6 > α ≥ 0.5 are poor, and lesser than 0.5 are unacceptable^37^.

Overall, the tool can be applicable to other areas or situations outside the northwest Ethiopian context which have similar characteristics with the study populations of the current study, such as rural settings in developing countries where the population has no access to improved WASH services or areas where the households are not linked to municipal water and sewage system. However, the generalizability of the tool to urban settings may be affected since access to WASH services in urban and rural settings are different.

### Limitation of the study

We initially planned to conduct construct validity and test–retest reliability. However, we didn’t do these since the variables were not factorable for factor analysis to test construct validity and the score of some variables are not stable over time, for instance WASH behavior or practice questions in the second survey were affected by the scores in the first survey.

## Conclusion

In this study, we designed valid and reliable questionnaire to assess behaviors and environmental conditions that result risk of exposure to enteric infections in rural settings. The items included in the questionnaire were found to be appropriate to assess individual behaviors and environmental conditions that result a high risk of exposure to enteric infections. The questionnaire can be used as a tool in the rural settings of developing countries with some amendments to account local contexts. However, this questionnaire alone does not measure exposure of children to enteric infections. It only complements external and internal exposure assessments. External exposure assessment (by identifying indicator organisms or specific pathogens in environmental samples using culture-dependent or culture-independent methods, molecular methods such as polymerase chain reaction (PCR) based assays, metagenomics to sequence and analyze all DNA in environmental samples, and biosensors) and internal exposure assessment, i.e., measuring enteropathogens in humans (using microscopy, enzyme-linked immunosorbent assays, PCR based assays, metagenomics, and pathogen-specific immunoassays) should be done to completely measure exposures to enteric infections as discussed by Goddard et al.^36^.

## Supplementary Information

Supplementary Information 1.

Supplementary Information 2.

## Data Availability

Data will be made available upon requesting the primary author.

## Abbreviations

CVR: Content validity ratio
I-CVI: Item-level content validity index
PCR: Polymerase chain reaction
S-CVI/UA: Universal agreement scale-level content validity index
WASH: Water, sanitation and hygiene
