# Supplementary material for: Development and validation of questionnaire to assess exposure of children to enteric infections in the rural northwest Ethiopia
Source: Sci Rep. 2022 Apr 25;12:6740. doi: 10.1038/s41598-022-10811-x (PMC9039032; doi:10.1038/s41598-022-10811-x)
Supplement: Supplementary file 1 — Supplementary Information 1. [file 41598_2022_10811_MOESM1_ESM.docx]

**This questionnaire is designed to collect data on conditions resulting risk of exposure of children to enteric infections in the rural setting of east Dembiya district, northwest Ethiopia**

**Questionnaire Identification Code: ______________________________________________**

**Consent Form**

Hello, I am ________________________ working with a research team from University of Gondar. We are contacting you to collect information for a study conducted to ***“*assess behaviors and environmental conditions resulting risk of child exposure to enteropathogens in the rural setting of northwest Ethiopia”**. This questionnaire is prepared to collect information from household, caregivers of children under five- years old and from children about personal hygiene, waste management, water quality and safety measures, food hygiene and safety measures, housing sanitation, infestation of vectors, child behaviors in relation to fecal-oral contamination, and access to health messages.

Thank you so much for agreeing to be interviewed for this project. All of the information we got from you will be completely confidential and coded with unique number. Your name will not be written down and will never be used in connection with any of the information you provide. There will not be any direct benefit that you will get associated with participation in this study. If you do not want to answer all or some of the questions, you have the right to refuse participation at any time. However, we would greatly appreciate your help in responding to this questionnaire. This questionnaire is expected to be completed in 30 minutes. If you have questions regarding your rights as a participant, you can discuss with the person lead researcher: Zemichael Gizaw, cell phone: +251913348400.

Do I have your permission to continue?

1. **Yes,** continue your interview with thanks after signing the consent

**______________________________________________________**

1. **If no,** skip to the next participant by writing reasons for his/ her refusal;

____________________________________________________________________________

**Data Collector:** Name: ____________________ Signature: ___________Date: __________

Time started: _____________________ Time completed: ____________________

| **Filled by field supervisors**  **Result of interview**:   1. Completed 2. Not completed 3. The respondent does not complete the whole questions   Name of Supervisor: ________________ Signature: ___________ Date: ________________ |
| --- |

| **Part 1: Socio-demographic information** | | | | | | | | | |
| --- | --- | --- | --- | --- | --- | --- | --- | --- | --- |
| 101 | Age of the mother or care giver | _______________________ | | | | | | | |
| 102 | Sex of the child | 1. Male 2. Female | | | | | | | |
| 103 | Age of the child | ____________________ | | | | | | | |
| 104 | Marital status of the mother or care giver | 1. Married 2. Single 3. Divorced 4. Separated 5. Widowed | | | | | | | |
| 105 | Educational status of the mother or care giver | 1. Can’t read and write 2. Can read and write 3. Primary school 4. Secondary school 5. Certificate or diploma 6. Degree | | | | | | | |
| 106 | If your response is “1” for question # 4, what is the education status of your spouse? | 1. Can’t read and write 2. Can read and write 3. Primary school 4. Secondary school 5. Certificate or diploma 6. Degree | | | | | | | |
| 107 | The highest level of education one or more of the family member has | 1. Can’t read and write 2. Can read and write 3. Primary school 4. Secondary school 5. Certificate or diploma 6. Degree | | | | | | | |
| 108 | Family size | ________________________ | | | | | | | |
| **Part 2: Health and sanitation information** | | | | | | | | | |
| 201 | Have you discussed about health, hygiene, sanitation and other health issues with the family members at regular basis? | 1. Yes 2. No | | | | | | | |
| 202 | Have you discussed about health, hygiene, sanitation and other health issues with the villagers? | 1. Yes 2. No | | | | | | | |
| 203 | Is there functional community health network in your area? | 1. Yes 2. No (go to # 205) | | | | | | | |
| 204 | If your answer is “1” for question # 203, are you or your family members actively participating? | 1. Yes 2. No | | | | | | | |
| 205 | Does the health extension worker or other health professional closely supervise you? | 1. Yes 2. No | | | | | | | |
| 206 | Have you heard any health/hygiene messages for the last 3 months? | 1. Yes 2. No ( go to # 208) | | | | | | | |
| 207 | If your answer is “Yes” for #206, which messages did you hear? | Wash hands | | Yes | | | | | No |
|  |  | Latrine utilization | | Yes | | | | | No |
|  |  | Drinking water quality management | | Yes | | | | | No |
|  |  | Food hygiene and safety | | Yes | | | | | No |
|  |  | Waste management | | Yes | | | | | No |
|  |  | Child feeding practice | | Yes | | | | | No |
|  |  | Child immunization | | Yes | | | | | No |
|  |  | Home remedies for common illnesses | | Yes | | | | | No |
| 208 | Are you practicing the messages listed in # 207? | 1. Yes (partly) 2. Yes (all) 3. No | | | | | | | |
| **Part 3: Personal hygiene** | | | | | | | | | |
| 301 | Kindly give me the key times you and other family members usually wash your hands? | Before eating | | Yes | | | | | No |
|  |  | After latrine use | | Yes | | | | | No |
|  |  | After handling baby’s diaper/feces | | Yes | | | | | No |
|  |  | After eating | | Yes | | | | | No |
|  |  | Before feeding child | | Yes | | | | | No |
|  |  | Before food preparation | | Yes | | | | | No |
|  |  | After handling rubbish | | Yes | | | | | No |
|  |  | After handling animals | | Yes | | | | | No |
| 302 | What do you usually use in washing hands? (more than one answer is possible) | 1. Water only 2. Water & Soap 3. Water & Sand/ soil 4. Water & Leaves 5. Water and ash | | | | | | | |
| 303 | Can you show me how you wash your hands? (interviewer: please check the following) | 1. Does the person use soap? | Yes | | | | | No | |
|  |  | 1. Does the person use ash or soil? | Yes | | | | | No | |
|  |  | 1. Does the person rub both hands together for at least 20 seconds | Yes | | | | | No | |
|  |  | 1. How does the person dry his/her hands | 1. Use their close 2. Dry in the air | | | | | | |
| 304 | Children frequently wash their hands after playing | 1. Yes 2. No | | | | | | | |
| 305 | Children frequently wash their hands after defecation | 1. Yes 2. No | | | | | | | |
| 306 | Children frequently wash their hands before eating | 1. Yes 2. No | | | | | | | |
| 307 | Is there any handwashing facility made from locally available materials? | 1. Yes 2. No (go to #309) | | | | | | | |
| 308 | If your answer is “Yes” for #307, observe | The container is filled with water | | | | Yes | | No | |
|  |  | The area is wet | | | | Yes | | No | |
|  |  | Soap or ash is available | | | | Yes | | No | |
| 309 | Does a caregiver or a mother keep her finger nails short and clean? | 1. Yes 2. No | | | | | | | |
| 310 | Does a child keep his/her finger nails short and clean? | 1. Yes 2. No | | | | | | | |
| 311 | Does the child mouth objects (e.g., fruit and food waste, cloth objects, non-cloth objects included plastic and metal objects, paper, wood furniture, glass, clay, bricks/concrete, and plant materials , and soil)  **Observe the child throughout the data collection time** | 1. Yes 2. No | | | | | | | |
| 312 | Does the child mouth his/her fingers? **Observe the child throughout the data collection time** | 1. Yes 2. No | | | | | | | |
| **Part 4: Excreta management including human feaces** | | | | | | | | | |
| 401 | Defecation practice of household members? (observe if it is latrine) | 1. Open field ( go to # 407) 2. Sanitary latrine 3. Both | | | | | | | |
| 402 | If it is sanitary latrine for # 401, what kind of toilet facility does this household use? | 1. Pit latrine 2. VIP latrine 3. Trench latrine | | | | | | | |
| 403 | If it is sanitary latrine for # 401, where is the toilet facility located? | 1. Inside or attached to dwelling 2. Elsewhere on premises 3. Outside premises | | | | | | | |
| 404 | If it is sanitary latrine for # 401, is the facility cleaned? | 1. Yes 2. No | | | | | | | |
| 405 | If it is sanitary latrine for # 401, does the squat hole have cover with handling? | 1. Yes 2. No | | | | | | | |
| 406 | If it is sanitary latrine for # 401, is there a visible sign of flies in or around the latrine? **(Observe it**) | 1. Yes 2. No | | | | | | | |
| 407 | How the household manage rubbish? | 1. Open dumping 2. Burning 3. Burying | | | | | | | |
| 408 | What does your household usually do with domestic wastewater (for example, from bathing, cleaning)? | 1. Use to water crops grown for livestock fodder 2. Use to water vegetable garden 3. Discard into soak pit or absorption pit 4. Splash everywhere in the premises | | | | | | | |
| 409 | Is the living environment is free from human feaces? | 1. Yes 2. No | | | | | | | |
| 410 | Is the living environment is free from animal feaces or chicken droppings? | 1. Yes 2. No | | | | | | | |
| 411 | Sanitation status of the village | 1. Non-open defecation free 2. Open defecation free | | | | | | | |
| 412 | Does the public open space free from human and animal feaces? | 1. Yes 2. No | | | | | | | |
| **Part 5: Water quality and safety measures** | | | | | | | | | |
| 501 | What is the main source of water for your household? | Protected well water | | | | Yes | | | No |
|  |  | Protected spring | | | | Yes | | | No |
|  |  | Protected rain catchment | | | | Yes | | | No |
|  |  | Unprotected well water | | | | Yes | | | No |
|  |  | Unprotected spring water | | | | Yes | | | No |
|  |  | Unprotected rain catchment | | | | Yes | | | No |
|  |  | Surface water (river, dam, lake, ponds, etc.) | | | | Yes | | | No |
| 502 | Does your water source provide water throughout the year? | 1. Yes 2. No | | | | | | | |
| 503 | How far from your dwelling the source of your drinking water? (in meter) | _________________________________ | | | | | | | |
| 504 | Time taken to fetch water (round trip and not including queuing time) in minute? | _______________________________ | | | | | | | |
| 505 | Queuing time in minute | __________________________________ | | | | | | | |
| 506 | How many liters of water do the entire households consume/use per day? (1 Jerry Can = 20L) | _________________________ | | | | | | | |
| 507 | What specific kind of water container do you use most to store water? (more than one answer is possible) | 1. Narrow mouthed containers like Jerry Can 2. Wide mouthed containers like clay pots, barrel 3. Both narrow and wide mouthed containers | | | | | | | |
| 508 | How do you usually withdraw (get) drinking water from the wide-mouthed container/storage? (more than one answer is possible) | 1. Tilt & pour into a cup/mug 2. Using tape mounted at the lower part of the containers 3. Dip separate mug/ cup | | | | | | | |
| 509 | Are the water storage containers clean? | 1. Yes 2. No | | | | | | | |
| 510 | Are the water storage containers are properly covered at the time of the survey? | 1. Yes 2. No | | | | | | | |
| 511 | Is the area where water storage containers places clean? | 1. Yes 2. No | | | | | | | |
| 512 | Is the water turbid? | 1. Yes 2. No | | | | | | | |
| 513 | Does the water have taste? | 1. Yes 2. No | | | | | | | |
| 514 | Do you practice homebased water treatment? | 1. Yes 2. No ( go to #601) | | | | | | | |
| 515 | If your answer is ‘Yes’ for question #514, which treatment do you use regularly? (more than one answer is possible) | Solar disinfection(SODIS) | | | Yes | | | No | |
|  |  | Chlorine/water guard/aqua tab/bleach | | | Yes | | | No | |
|  |  | Boiling | | | Yes | | | No | |
|  |  | Cloth filtration | | | Yes | | | No | |
|  |  | Plain sedimentation | | | Yes | | | No | |
| **Part 6: Food hygiene and safety measures** | | | | | | | | | |
| 601 | Are you practicing the following when you prepared foods? | Wash hands before preparation | | | Yes | | | No | |
|  |  | Wash utensils and containers before preparation | | | Yes | | | No | |
|  |  | Proper washing of foods that should be washed | | | Yes | | | No | |
|  |  | Thorough cooking of foods that should be cooked | | | Yes | | | No | |
|  |  | Cover hair | | | Yes | | | No | |
|  |  | Don’t touch my body parts and other things | | | Yes | | | No | |
| 602 | Do you prepare food while you have diarrhea/or vomiting or other communicable diseases? | 1. Yes 2. No | | | | | | | |
| 603 | Do you use separate cutting board or knife or utensils for cooked and raw foods? | 1. Yes 2. No | | | | | | | |
| 604 | What do you use to wash food utensils? | 1. Water only 2. Soap and water 3. Ash and water | | | | | | | |
| 605 | How do you dry washed food utensils? | 1. Perforated drain board or rack 2. Wipe with cloth 3. Dry in the air | | | | | | | |
| 606 | Where you store foods and food utensils? | 1. On the floor 2. In shelf made from wood and mud | | | | | | | |
| 607 | Do you use leftover foods? | 1. Yes 2. No | | | | | | | |
| 608 | If your answer is “yes” for question #607, do you properly reheat it? (proper reheating is cooking foods for at least 5 minutes after steam is created) | 1. Yes 2. No | | | | | | | |
| 609 | Observe the condition of food utensils | Are clean | | | | | Yes | | No |
|  |  | Are covered properly | | | | | Yes | | No |
|  |  | Are properly stored in clean area or shelf | | | | | Yes | | No |
|  |  | Are not protected from pets | | | | | Yes | | No |
| 610 | Vectors or rodents are seen in food storage area | 1. Yes 2. No | | | | | | | |
| 611 | Condition of kitchen | 1. Clean 2. Not clean (has visible dirt) | | | | | | | |
| **Part 7: Housing and living environment sanitation** | | | | | | | | | |
| 701 | Number of rooms | __________________ | | | | | | | |
| 702 | Do you clean the internal and external areas of the house regularly at least once a week | 1. Yes 2. No | | | | | | | |
| 703 | Cleanliness of the building (observe the building and judge based on the checklist) | 1. Clean   Not clean (the floor, wall, or ceiling has visible dirt) | | | | | | | |
| 704 | Where domestic animals are kept? | 1. No domestic animals 2. In barn constructed attached with the main building 3. Separately constructed animal shed (barn) 4. In the same house with human beings. 5. Open field | | | | | | | |
| 705 | Is the living compound free from human or animal feaces (observe the yard and judge based on the checklist) | 1. Yes 2. No | | | | | | | |
| 706 | Is the living compound free from garbage? (observe the yard and judge based on the checklist) | 1. Yes 2. No | | | | | | | |
| 707 | Is the living compound free from sewage? (observe the yard and judge based on the checklist) | 1. Yes 2. No | | | | | | | |
| 708 | Is there human and animal excreta in child playing areas? (observe the area and judge based on the checklist) | 1. Yes 2. No | | | | | | | |
| **Part 8: Enteric infections** | | | | | | | | | |
| 801 | Has the child had diarrhea during the past 24 hours? (A child is considered having diarrhea if he/ she defecated three or more liquid stools in a 24-h period) | 1. Yes 2. No | | | | | | | |
| 802 | Has the child had diarrhea in the last 2 weeks? | 1. Yes 2. No | | | | | | | |
| 803 | Did the stool contain (checked by field data collectors) | 1. Blood 2. Mucus 3. Don’t know | | | | | | | |
| 804 | Did you seek medical advice or treatment for the diarrhea? | 1. Yes 2. No | | | | | | | |
| 805 | Do the child has intestinal parasites | 1. No 2. Single infection 3. Multiple infections 4. I don’t know | | | | | | | |
| 806 | Do you think diarrhea or intestinal parasitic infections are communicable? | 1. Yes 2. No | | | | | | | |

**We finished our survey. Thank you for your participation**.
