# Supplementary material for: Development and validation of questionnaire to assess exposure of children to enteric infections in the rural northwest Ethiopia
Source: Sci Rep. 2022 Apr 25;12:6740. doi: 10.1038/s41598-022-10811-x (PMC9039032; doi:10.1038/s41598-022-10811-x)
Supplement: Supplementary file 2 — Supplementary Information 2. [file 41598_2022_10811_MOESM2_ESM.pdf]

ይህ መጠይቅ በምስራቅ ደምቢያ ወረዳ ገጠራማ አካባቢዎች የሚኖሩ እድሜአቸው ከ 5 አመት በታች የሆኑ ህጻናትን ለበሽታ የሚያጋልጧቸውን ነገሮችንና ተያያዥ ጉዳዮችን ለመዳሰስ የተዘጋጀ ነው፡፡

የመጣይቁ መለያ ኮድ፡______________________________________

በጥናቱ ለመሳተፍ የስምምነት መጠየቂያ ቅጽ

ሰላም ጤና ይስጥልኝ፡፡ እኔ ___________________________ እባላለሁ፡፡ የመጣሁት የጎንደር ዩኒቨርሲቲ መምህራን ለሚያካሂዱት ጥናት መረጃ ለመሰብሰብ ነው፡፡ ጥናቱ በዋናነት በምስራቅ ደምቢያ ወረዳ ገጠራማ አካባቢዎች የሚኖሩ እድሜአቸው ከ 5 አመት በታች የሆኑ ህጻናትን ለበሽታ የሚያጋልጧቸውን ነገሮችንና ተያያዥ ጉዳዮችን ለማዎቅ ያለመ ነው፡፡

በጥናቱ ለመሳትፍ ስለተስማሙ እናሰግናለን፡፡ ከርስዎ የምናገኘው መረጃ ሚስጢራዊነቱ የተጠበቀ ይሆናል፡፡ በመጠይቁ ስምዎ አይጠቀስም፡፡ በጥናቱ ስለተሳተፉ ቀጥተኛ ጥቅም አያገኙም፡፡ ጥያቄዎቻችንን መጠየቅ የምንችለው የርስዎን ፍቃድ ስናገኝ ነው፡፡ በዚህ ጥናት መሳተፍ ካልፈለጉ አለመሳተፍ ይችላሉ፡፡ ሁሉንም ጥያቄዎች የመመለስ ግደታ የለብዎትም፡፡ መጠይቁን በፈለጉት ጊዜ ማቋረጥ ይችላሉ፡፡ነገር ግን ሁሉንም ጥያቄዎች ቢመልሱና በመሃል ባያቋርጡ የምንፈልገውን መረጃ እንድናገኝ ይረዳናል፡፡ መጠይቁን በ 30 ደቂቃ ውስጥ እንጨርሳለን፡፡ ስለ ጥናቱና ስለ መብትዎ ጥያቄ ካለዎት ከጥናቱ ዋና አስተባባሪ ከአቶ ዘሚካኤል ግዛው ጋር መዎያየት ይችላሉ ፡፡ የዘሚካኤልን ስልክ ቁጥር ከፈለጋችሁ ስልክ ቁጥሩ +251913348400 ነው፡፡

በጥናቱ ለመሳተፍ ፈቃደኛ ነዎት? ፈቃደኛ ከሆኑ ጥያቄዎችን መጠየቅ እችላለሁ?

1. አዎ ፈቃደኛ ነኝ፡፡ ፈቃደኛነትዎትን ከታች በተዘጋጀው ቦታ በመፈረም ያረጋግጡልን፡፡

   ____

2. ፈቃደኛ አይደለሁም፡፡ ፈቃደኛ ካልሆኑ ምክንያታቸውን ከታች በተዘጋጀው ቦታ በመጻፍ ወደ ቀጣይ ተሳታፊ ይለፉ፡፡

____

የመረጃ ሰብሳቢው ስም: _______________________ ፊርማ: ____________ቀን: ___________

መጠይቁ የተጀመረበት ሰአት: _______________ መጠይቁ የተጠናቀቀበት ሰአት: __________________

በመስክ ተቆጣጣሪዎች የሚሞላ

የመጠይቁ ሁኔታ

1. ሙሉ በሙሉ ተጠናቋል
2. ሙሉ በሙሉ አልተጠናቀቀም
3. የመጠይቁ ተሳታፊ ጥያቄዎችን ሳይጨርሱ አቋርጠዋል፡፡

የመስክ ተቆጣጣሪ ስም: ______________________ ፊርማ: _____________ ቀን: __________________

ክፍል 1፡ ስነ-ህዝባዊ/ ኢኮኖሚያዊ መረጃዎች		
101	የእናት ወይም ያሳዳጊ እድሜ	
102	የህጻኑ/ኗ ጾታ	
103	የህጻኑ/ኗ እድሜ	
104	የእናት ወይም ያሳዳጊ የጋብቻ ሁኔታ	1. ያገባ/ች<br>2. ያላገባ/ች<br>3. የፈታ/ች<br>4. ተለያይተው የሚኖሩ<br>5. ባል/ሚስት የሞተባት/የሞተችበት
105	የእናት ወይም ያሳዳጊ የትምህርት ደረጃ	1. ማንበብና መጻፍ የማይችሉ<br>2. ማንበብና መጻፍ የሚችሉ<br>3. የመጀመሪያ ደረጃ ትምህርት<br>4. የሁለተኛ ደረጃ ትምህርት<br>5. ሰርቲፊኬት/ድፕሎማ<br>6. ድግሪ
106	ለጥያቄ #104 መልስዎ 1 ከሆነ የትዳር አጋርዎ የትምህርት ደረጃ	1. ማንበብና መጻፍ የማይችሉ<br>2. ማንበብና መጻፍ የሚችሉ<br>3. የመጀመሪያ ደረጃ ትምህርት<br>4. የሁለተኛ ደረጃ ትምህርት<br>5. ሰርቲፊኬት/ድፕሎማ<br>6. ድግሪ
107	ቤተሰቡ ውስጥ ከፍተኛ የትምህርት ደረጃ የትኛው ነው?	1. ማንበብና መጻፍ መቻል<br>2. የመጀመሪያ ደረጃ ትምህርት<br>3. የሁለተኛ ደረጃ ትምህርት<br>4. ሰርቲፊኬት/ድፕሎማ<br>5. ድግሪ
108	የቤተሰብ ብዛት	---

ክፍል 2: የጤናና ንጽህና መረጃዎች				
201	የቤተሰቡ አባላት ስለ ጤና (ለምሳሌ ግልና አካባቢ ንጽህና እና ተላላፊ በሽታዎች) ቀጣይነት ባለው መልኩ ይዎያያሉ?	1. እንዎያያለን<br>2. አንዎያይም		
202	ከጎረቢት/ ዎይም ከሌሎች የሰፈሩ ሰዎች ስለ ጤና፣ ግልና አካባቢ ንጽህና ቢያንስ በወር አንድ ጊዜ ቀጣይነት ባለው መልኩ ይዎያያሉ?*	1. እንዎያያለን<br>2. አንዎያይም		
203	በአካባቢያችሁ አገልግሎት የሚሰጥ የጤና ቡድን አለ?*	1. አዎ 2. የለም (ወደ #205 እለፍ)		
204	የጤና ቡድን ካለ እርስዎ ዎይም ሌሎች የቤተሰብ አባላት በንቃት ይሳተፋሉ?	1. አዎ 2. የለም		
205	የጤና ኤክስቴንሽን ወይም የጤና ቡድኑ ቢያንስ በወር አንደ ቀጣይነት ባለው መልኩ ቁጥጥርና እገዛ ያደርጉላችኋል?	1. አዎ<br>2. አይደለም		
206	ባለፉት 3 ወራት ስለ ጤና (ለምሳሌ ግልና አካባቢ ንጽህና እና ተላላፊ በሽታዎች) መረጃ/ የጤና ትምህርት አግኝተው ያውቃሉ?	1. አግኝቼ አውቃለሁ<br>2. አግኝቼ አላውቅም (ወደ #208 እለፍ)		
207	ለጥያቄ ቁጥር 206 መልስዎ መረጃ አግኝቼ አውቃለሁ ከሆነ ምን አይነት መረጃ ነው ያገኙት?	ስለ እጅ አስተጣጠብ	አዎ	የለም
		ስለሽንት ቤት አጠቃቀም	አዎ	የለም
		ስለ መጠጥ ውሃ ንጽህና አጠባበቅና አያያዝ	አዎ	የለም
		ስለ ምግብ ንጽህና አጠባቅና አያያዝ	አዎ	የለም
		ስለ ቆሻሻ አዎጋገድ	አዎ	የለም
		ስለ ህጻናት አመጋገብ፣ ስነ-ምግብና ስለ እናት ጡት ጥቅም	አዎ	የለም
		ስለ ክትባት	አዎ	የለም
		በሽታ በሚይዘን ወቅት ቤት ውስጥ ማድረግ ስለሚገባን	አዎ	የለም

<table>
<tr><td></td><td></td><td colspan="2">ጥንቃቄ</td><td></td><td></td></tr>
<tr><td>208</td><td>ከላይ በጥያቄ ቁጥር 207 የዘረዘሯቸውን የጤና መረጃዎች እየተገበሯቸው ነው?</td><td colspan="4">1. በከፊል እየተገበርኩ ነው
2. ሙሉ በሙሉ እየተገበርኩ ነው
3. እየተገበርኩ አይደለም</td></tr>
<tr><td colspan="6">ክፍል 3: ስለ ግል ንጽህና መረጃ</td></tr>
<tr><td rowspan="8">301</td><td rowspan="8">አንተ/ቺ ወይም ሌሎች የቤተሰብ አባላት እጃችሁን በአብዛኛው መቼ እንደምትጣጠቡ ሊነግሩኝ ይችላሉ?</td><td colspan="2">ምግብ ከመመገባችን በፊት</td><td>አዎ</td><td>የለም</td></tr>
<tr><td colspan="2">ከሽንት ቤት መልስ</td><td>አዎ</td><td>የለም</td></tr>
<tr><td colspan="2">ህጻናትን ካጸዳዳን በኋላ</td><td>አዎ</td><td>የለም</td></tr>
<tr><td colspan="2">ምግብ ከተመገብን በኋላ</td><td>አዎ</td><td>የለም</td></tr>
<tr><td colspan="2">ህጻናትን ከመመገባችን በፊት</td><td>አዎ</td><td>የለም</td></tr>
<tr><td colspan="2">ምግብ ከማዘጋጀታችን በፊት</td><td>አዎ</td><td>የለም</td></tr>
<tr><td colspan="2">ቆሻሻ ከነካን በኋላ</td><td>አዎ</td><td>የለም</td></tr>
<tr><td colspan="2">እንስሳትን ከነካን በኋላ</td><td>አዎ</td><td>የለም</td></tr>
<tr><td>302</td><td>እጅዎትን ሲታጠቡ አብዛኛውን ጊዜ ምን ይጠቀማሉ? (ከአንድ በላይ መልስ ይቻላል)</td><td colspan="4">1. ውሃ ብቻ
2. ውሃና ሳሙና
3. ዉሃና አፈር
4. ውሃና ቅጠል
5. ውሃና አመድ</td></tr>
<tr><td rowspan="4">303</td><td rowspan="4">እጅዎትን እንዴት እንደሚታጠቡ ሊያሳዩኝ ይችላሉ?</td><td colspan="2">1. ሲታጠቡ ሳሙና ተጠቅመዋል</td><td>አዎ</td><td>አይደለም</td></tr>
<tr><td colspan="2">2. ሲታጠቡ አፈር/ አመድ ተጠቅመዋል</td><td>አዎ</td><td>አይደለም</td></tr>
<tr><td colspan="2">3. ሲታጠቡ ሁሉንም የእጃቸውን ክፍል ቢያንስ ለ 20 ሴኮንድ አሸተዋል</td><td>አዎ</td><td>አይደለም</td></tr>
<tr><td>4. ከታጠቡ በኋላ እጃቸውን በምን አደረቁ?</td><td colspan="3">1. ልብሳቸው ላይ በመጠራረግ
2. በአየር ላይ እንድደርቅ አድርገዋል</td></tr>
<tr><td>304</td><td>ህጻናትን ከጫዎታ መልስ እጃቸውን ሁልጊዜ ያጥቧቸዋል?</td><td colspan="4">1. አዎ 2. አይደለም</td></tr>
<tr><td>305</td><td>ህጻናት ከተጸዳዱ በኋላ እጃቸውን ሁልጊዜ ያጥቧቸዋል?</td><td colspan="4">1. አዎ 2. አይደለም</td></tr>
<tr><td>306</td><td>ህጻናትን ማንኛውንም ምግብ ከመመገባቸው በፊት እጃቸውን ሁልጊዜ ያጥቧቸዋል</td><td colspan="4">1. አዎ
2. አይደለም</td></tr>
<tr><td>307</td><td>አገልግሎት የሚሰጥ የእጅ መታጠቢያ አለ?</td><td colspan="4">1. አለ 2. የለም (ወደ #309 እለፍ)</td></tr>
<tr><td rowspan="3">308</td><td rowspan="3">ለጥያቄ #307 መልሱ አለ ከሆነ</td><td colspan="2">የእጅ መታጠቢያው ውሃ ተሞልቷል</td><td>አዎ</td><td>አይደለም</td></tr>
<tr><td colspan="2">አካባቢው ረጥቧል</td><td>አዎ</td><td>አይደለም</td></tr>
<tr><td colspan="2">ሳሙና ወይም አመድ በአካባቢው አለ</td><td>አዎ</td><td>አይደለም</td></tr>
<tr><td>309</td><td colspan="2">የእናት ወይም የህጻናት አሳዳጊ የእጅ ጥፍር በአጭሩ የተቆረጠና ንጽህ ነው? (እጃቸውን ይመልከቱ)</td><td colspan="3">1. አዎ 2. አይደለም</td></tr>
<tr><td>310</td><td colspan="2">የህጻናት የእጅ ጥፍር በአጭሩ የተቆረጠና ንጽህ ነው? (እጃቸውን ይመልከቱ)?</td><td colspan="3">1. አዎ 2. አይደለም</td></tr>
<tr><td>311</td><td colspan="2">ህጻናት ያገኙትን ማንኛውንም ነገር (ለምሳሌ ልብሳቸውን፣ እንጨት፣ አፈር፣ ወዘተ) ወደ አፋቸው ያስገባሉ?
ካሁን በፊት የነበራቸውን ተግባር ወላጆቻቸውን ጠይቅ/ቂ ፡፡ በተጨማሪም ግቢ ወይም ቤት ውስጥ ከገቡ ጀምሮ መጠይቁን አጠናቀው እስከሚወጡ ድረስ የህጻናትን ሁኔታ ተከታተል/ይ</td><td colspan="3">1. አዎ
2. አይደለም</td></tr>
<tr><td>312</td><td colspan="2">ህጻናት ጣታቸውን ወደ አፋቸው ያስገባሉ?
ካሁን በፊት የነበራቸውን ተግባር ወላጆቻቸውን ጠይቅ/ቂ ፡፡ በተጨማሪም ግቢ ወይም ቤት ውስጥ ከገቡ ጀምሮ መጠይቁን አጠናቀው እስከሚወጡ ድረስ የህጻናትን ተከታተል/ይ</td><td colspan="3">1. አዎ
2. አይደለም</td></tr>
<tr><td colspan="6">ክፍል 4: የቆሻሻ አያያዝና አዎጋገድን የሚመለከት መረጃ</td></tr>
<tr><td>401</td><td>የቤተሰቡ አባላት የሚጸዳዱት የት ነው?</td><td colspan="4">1. ሜዳ ላይ (ወደ ጥያቄ #407 እለፍ/ፊ)</td></tr>
</table>

		2. ሽንት ቤት<br>3. ሁለቱንም እንጠቀማለን
402	ለጥያቄ ቁጥር 401 መልሱ ሽንት ቤት ከሆነ ሽንት ቤቱ ምን አይነት ነው?	1. ርብራብና መጠለያ ያለው ሽንት ቤት<br>2. ርብራብ፣ መጠለያ ና ማስተንፈሻ ቱቦ ያለው ሽንት ቤት<br>3. ርብራብና መጠለያ የሌለው ጉድጓድ
403	ለጥያቄ ቁጥር 401 መልሱ ሽንት ቤት ከሆነ ሽንት ቤቱ የት ነው የተሰራው?	1. ከመኖሪያ ቤት ጋር ተያይዞ<br>2. በግቢ ውስጥ<br>3. ከግቢ ውጭ
404	ለጥያቄ ቁጥር 401 መልሱ ሽንት ቤት ከሆነ ሽንት ቤቱና አካባቢው ከሰገራ የጸዳ ነው? (አካባቢውን ተመልከት/ች)	1. አዎ<br>2. አይደለም
405	ለጥያቄ ቁጥር 401 መልሱ ሽንት ቤት ከሆነ የሽንት ቤቱ ቀዳዳ መክደኛ አለው? (ሽንት ቤቱን በመመልከት ይመልሱ)	1. አዎ<br>2. አይደለም
406	ለጥያቄ ቁጥር 401 መልሱ ሽንት ቤት ከሆነ በሽንት ቤቱና አካባቢው ዝንቦች ይታያሉ? (ሽንት ቤቱንና አካባቢውን በመመልከት ይመልሱ)	1. አዎ<br>2. አይደለም
407	ደረቅ ቆሻሻዎችን እንዴት ነው የምታስወግዱት?	1. ሜዳ ላይ መበተን<br>2. ማቃጠል<br>3. መቅበር
408	የፍሳሽ ቆሻሻን እንዴት ነው የምታስዎግዱት?	1. ለቤት እንስሳት መኖ የሚሆኑ እንደ ሳር ወይም አዝዕርት ለማብቀያነት እንጠቀማለን<br>2. ለጓሮ አትክልት ማልሚያ እንጠቀማለን<br>3. የፍሳሽ ቆሻሻ ማስረጊያ በመጠቀም ማስዎገድ<br>4. በተገኘው ቦታ/ሜዳ ላይ ማፍሰስ
409	የመኖሪያ አካባቢው ከሰው ጽዳጅ የጸዳ ነው? (አካባቢውን ይመልከቱ)	1. አዎ 2. አይደለም
410	የመኖሪያ አካባቢው ከእንስሳት ጽዳጅ ወይም ከዶሮ ኩስ የጸዳ ነው? (አካባቢውን ይመልከቱ)	1. አዎ 2. አይደለም
411	የሰፈሩ ሁኔታ*	1. በየሜዳው ከመጸዳዳትና ቆሻሻ ከመጣል የጸዳ ተብሎ የተሰየመ (open defecation free)<br>2. በየሜዳው ከመጸዳዳትና ቆሻሻ ከመጣል ያልጸዳ
412	የሰፈሩ የጋራ ቦታ ከሰውና ከእንስሳት ጽዳጅ ወይም ከቆሻሻ የጸዳ ነው?*	1. አዎ 2. አይደለም

ክፍል 5: የመጠጥ ውሃ ንጽህናን የሚመለከት መረጃ

501	ቤተሰቡ የመጠጥ ውሃ የሚያገኘው ከዬት ነው?*	ከሰው ወይም ከእንስሳት ንክኪ፤ ከጎርፍና ከንፋስ ከተጠበቀ የጉድጓድ ውሃ	አዎ	የለም
		ከሰው ወይም ከእንስሳት ንክኪ፤ ከጎርፍና ከንፋስ ከተጠበቀ የምንጭ ውሃ	አዎ	የለም
		ከሰው ወይም ከእንስሳት ንክኪ፤ ከጎርፍና ከንፋስ ከተጠበቀ የዝናብ ውሃ ማጠራቀሚያ ገንዳ	አዎ	የለም
		ከሰው ወይም ከእንስሳት ንክኪ፤ ከጎርፍና ከንፋስ ካልተጠበቀ የጉድጓድ ውሃ	አዎ	የለም
		ከሰው ወይም ከእንስሳት ንክኪ፤ ከጎርፍና ከንፋስ ካልተጠበቀ የምንጭ ውሃ	አዎ	የለም
		ከሰው ወይም ከእንስሳት ንክኪ፤ ከጎርፍና ከንፋስ ካልተጠበቀ የዝናብ ውሃ ማጠራቀሚያ ገንዳ	አዎ	የለም
		ከወንዝ፣ ከግድብ ወይም ከኩሬ	አዎ	የለም
502	ዉሃ አመቱን ሙሉ ሳይቋራረጥ ያገኛሉ?*	1. አገኛለሁ 2. አላገኝም		
503	የመጠጥ ውሃ የሚያገኙበት ቦታ ከቤትዎ ምን ያክል ይርቃል?	____________________ ሜትር		
504	ውሃ ቀድቶ ለመመለስ ምንያክል ደቂቃ ይወስድብዎታል?	____________________		

505	ውሃ ለማግኘት ምን ያክል ደቂቃዎች ወረፋ ይጠብቃሉ?	______________________
506	በቀን ምን ያክል ሊትር ውሃ ይቀዳሉ? (1 ጀሪካን = 20 ሊትር)	____________________________
507	ውሃ የሚቀዱት ወይም በቤት ውስጥ ውሃ በምንድን ነው የሚያጠራቅሙት?	1. በአፈ ጠባብ እቃ ለምሳሌ ጀሪካን<br>2. በአፈ ሰፊ እቃ ለምሳሌ እንስራ ፣ የፕላስቲክ በርሜል<br>3. ሁለቱንም አይነት እቃዎች እንጠቀማለን
508	ውሃ በአፈ ሰፊ እቃ የሚያስቀምጡ ከሆነ ውሃ ለመጠቀም እንደት ነው የሚቀዱት? (ከአንድ በላይ መልስ ይቻላል)	1. የማጠራቀሚያ እቃውን በማዘንበል ወደ ሌላ እቃ መቅዳት<br>2. ከማጠራቀሚያ እቃው ታችኛ ክፍል በተዘጋጀ የቧንቧ መክፈቻና መዝጊያ በመጠቀም<br>3. መጥለቂያ እቃ በመጠቀም መቅዳት
509	የውሃ መቅጃ ወይም ማጠራቀሚያ እቃዎች ንጹህ ናቸው? (እቃዎችን ይመልከቱ)	1. አዎ 2. አይደሉም
510	መረጃ በሚሰበስቡበት ጊዜ የውሃ መቅጃ ወይም ማጠራቀሚያ እቃዎች ተከድነዋል? (እቃዎችን ይመልከቱ)	1. አዎ<br>2. አልተከደኑም
511	የውሃ መቅጃ ወይም ማጠራቀሚያ እቃዎች የሚቀመጡበት ቦታ ንጹህ ነው? (አካባቢውን ይመልከቱ)	1. አዎ<br>2. አይደለም
512	ለመጠጥ የሚጠቀሙት ውሃ የደፈረሰ ነው (አፈር ወይም ሌሎች ነገሮች አሉት)?* (ውሃ በብርጭቆ አስቀድተው ይመልከቱ)	1. አዎ<br>2. አይደለም
513	ለመጠጥ የሚጠቀሙት ውሃ ጣእም አለው?*	1. አዎ 2. የለውም
514	የመጠጥ ውሃን በቤት ውስጥ ያክማሉ?	1. አዎ እናክማለን<br>2. አናክምም (ወደ ጥያቄ #601 እለፍ/ፊ)

515	የመጠጥ ውሃን በቤት ውስጥ የሚያክሙ ከሆነ የሚያክሙበት ዘዴ ምንድን ነው?	የጸሃይ ብርሃን በመጠቀም ውሃን ማከም	አዎ	የለም
		ክሎሪንና የክሎሪን ውጤቶችን (ለምሳሌ ውሃ አጋር) በመጠቀም ማከም	አዎ	የለም
		ማፍላት	አዎ	የለም
		የልብስ ማጥለያ መጠቀም	አዎ	የለም
		ማዝቀጥ	አዎ	የለም

ክፍል 6: የምግብ ንጽህናን የሚመለከት መረጃ

601	ምግብ ሲያዘጋጁ የሚከተሉትን ይተገብራሉ?	ከምግብ ዝግጅት በፊት እጅን በደንብ መታጠብ	አዎ	የለም
		የምግብ ማዘጋጃ እቃዎችን በደንብ ማጠብ	አዎ	የለም
		ታጥበው መዘጋጀት ያለባቸውን ምግቦች ማጠብ	አዎ	የለም
		የሚበስሉ ምግቦችን በደንብ ማብሰል	አዎ	የለም
		ጸጉርን መሸፈን	አዎ	የለም
		ሰውነቴንና ሌሎች ነገሮችን አለመነካካት	አዎ	የለም
602	የህመም ስሜት ለምሳሌ ተቅማጥ ወይም ትውከት እያለዎት ምግብ ያዘጋጃሉ?	1. አዎ 2. አይደለም		
603	ላልበሰሉና ለበሰሉ ምግቦች ማዘጋጃ የተለየ ቢለዋና መክተፊያ ይጠቀማሉ?	1. አዎ 2. አይደለም		
604	የምግብ እቃዎችን ለማጠብ ምን ይጠቀማሉ?	1. ውሃ ብቻ<br>2. ሳሙናና ውሃ<br>3. አመድና ውሃ		

605	የታጠቡ የምግብ እቃዎችን እንዴት ነው የሚያደርቁት?	1. ማንጠፍጠፊያ መጠቀም<br>2. በልብስ ወይም በሌላ መወልወያ መዎልወል<br>3. በአየር ላይ እንድደርቁ ማድረግ
606	የታጠቡ የምግብ እቃዎችን የት ነው የሚያስቀምጡት? (በተለያዩ ቦታዎች የሚያስቀምጡ ከሆነ ሁሉንም አረጋግጥ/ጭ)	1. ወለል ላይ<br>2. መደርደሪያ ላይ
607	የዋለ ወይም ያደረ ምግብ ለህጻናት ይሰጣሉ?	1. አዎ 2. አንሰጥም
608	የዋለ ወይም ያደረ ምግብ ለህጻናት ከሰጡ ከመስጠትዎ በፊት በደንብ ያሞቃሉ? (በደንብ ማሞቅ ማለት የሚሞቀው ምግብ እንፋሎት ከፈጠረ በኋላ ቢያንስ ለ 5 ደቂቃ ማሞቅ ወይመቀቀል ማለት ነው)	1. አዎ 2. አይደለም

609	ምግብ የያዙ የምግብ እቃዎችን ይመልከቱና የሚከተሉትን አረጋግጡ	ንጹህ ናቸው	አዎ	የለም
		በደንብ ተከድነዋል	አዎ	የለም
		በንጹህ ቦታ ዎይም መደርደሪያ ተቀምጠዋል	አዎ	የለም
		የቤት እንስሳት ሊያገኟቸው ይችላሉ	አዎ	የለም

610	ምግብና ምግብ ነክ ነገሮች በሚቀመጡበት ቦታ እንደ ዝንብ፣ በረሮና አይጥ ብዙ ጊዜ ይታያሉ	1. አዎ<br>2. አይታዩም
611	የምግብ ማዘጋጃ ቦታ ዎይም የኩሽናው የንጽህና ሁኔታ	1. ንጹህ ነው 2. ንጹህ አይደለም
**ክፍል 7: የመኖሪያ ቤትና አካባቢ ንጽህና የሚመለከት መረጃ**		
701	የመኖሪያ ቤቱ ስንት ክፍሎች አሉት?	____________________
702	የቤቱን ውስጣዊና ውጫዊ ክፍል ቢያንስ በሳምንት አንድ ጊዜ ቀጣይነት ባለው መልኩ ያጸዳሉ?	1. አዎ 2. የለም
703	የቤቱ የንጽህና ሁኔታ(የቤቱን ውስጣዊና ውጫዊ ክፍል በማየት ይሞላ):: የንጽህናውን ሁኔታ ለመወሰን የተዘጋጀውን ማብራሪያ ይጠቀሙ	1. ንጹህ ነው<br>2. ንጹህ አይደለም (ወለሉ ወይም ግድግዳው ወይም ጣራው ቆሻሻ ወይም ጥቀርሻ ወይም የሸረሪት ድር ወይም የነፍሳት ቅሪት አካል/ደም ካለው)
704	የቤት እንስሳት የሚኖሩት የት ነው?	1. የቤት እንስሳት የለንም<br>2. ከመኖሪያቤቱ ጋር ተያይዞ በተሰራ ጋጣ<br>3. ከመኖሪያ ቤቱ ተራርቆ በተሰራ ጋጣ<br>4. ከመኖሪያቤት ውስጥ<br>5. ከግቢ ውስጥ
705	ግቢው ከሰው ዎይም ከእንስሳት ጽዳጅ የጸዳ ነው? (ጊቢውን ዞር ዞር ብለህ/ሽ ተመልከት/ች)	1. አዎ 2. አይደለም
706	ግቢው ከደረቅ ቆሻሻ የጸዳ ነው? (ጊቢውን ዞር ዞር ብለህ/ሽ ተመልከት/ች)	1. አዎ 2. አይደለም
707	ግቢው ከፍሳሽ ቆሻሻ የጸዳ ነው? (ጊቢውን ዞር ዞር ብለህ/ሽ ተመልከት/ች)	1. አዎ 2. አይደለም
708	ህጻናት በሚጫዎቱበት ቦታ የሰው ዎይም የእንስሳት ጽዳጅ፣ ደረቅ ቆሻሻና ፍሳሽ ቆሻሻ ይታያል (ህጻናት የሚጫዎቱበትን ቦታ ዞር ዞር ብለህ/ሽ ተመልከት/ች)*	1. አዎ<br>2. አይደለም
**ክፍል 8: ተላላፊ በሽታዎችን የሚመለከት መረጃ**		
801	ህጻኑ/ኗ ባለፉት 24 ሰኣት ውስጥ የተቅማጥ በሽታ ነበው/ረባት? (ተቅማጥ ማለት ህጻኑ/ኗ በ 24 ሰኣት ውስጥ ሶስትና ከዚያ በላይ ቀጠን ያለ ሰገራ ከተጸዳዳ/ ከተጸዳዳች)	1. አዎ 2. የለም
802	ህጻኑ/ኗ ባለፉት 2 ሳምንታት ውስጥ የተቅማጥ በሽታ ነበው/ረባት	1. አዎ 2. የለም
803	የህጻኑ/ኗ ሰገራ (በመረጃ ሰብሳቢዎች የሚሞላ)	1. ደም የቀላቀለ ነው<br>2. ዝልግልግ ነገር አለው<br>3. አላውቅም
804	ህጻኑ/ኗ የተቅማጥ በሽታ ከነበው/ረባት ወደ ህክምና ወስደውት/ዋት ነበር	1. አዎ 2. የለም
805	ህጻኑ/ኗ የአንጀት ጥገኛ ትላትሎች አሉት/ አ□ት	1. የለም<br>2. አንድ የአንጀት ጥገኛ ትል አለው/ አ□ት

		3. ብዙ የአንጀት ጥገኛ ትላትል አለው/ አ□ት<br>4. አላውቅም
806	የተቅማጥ ወይም የአንጀት ጥገኛ ትላትል በሽታ ተላላፊ ነው ብለው ያስባሉ	1. አዎ 2. አይደለም

ጥያቄዎቹን ጨርሻለሁ። ስለተሳትፎዎ ከልብ እናመሰግናለን።
